# Pulmonary development in Squamata: Insights from embryonic studies using micro‐CT

**DOI:** 10.1002/dvdy.70062

**Published:** 2025-07-16

**Authors:** Barbara G. Champini, Raul E. Diaz, Emma R. Schachner, Wilfried Klein

**Affiliations:** ^1^ Departamento de Biologia, Faculdade de Filosofia, Ciências e Letras de Ribeirão Preto Universidade de São Paulo Ribeirão Preto São Paulo Brazil; ^2^ Department of Biological Sciences California State University Los Angeles California USA; ^3^ Department of Physiological Sciences, College of Veterinary Medicine University of Florida Gainesville Florida USA

**Keywords:** computed tomography, embryology, non‐invasive technique, reptiles

## Abstract

**Background:**

Pulmonary development in tetrapods is a complex process, especially within squamates, where single‐chambered, transitional, and multi‐chambered lungs can be found in adult animals. While the embryological development of the respiratory system of lizards and snakes was studied in a number of species between the 1830s and 1940s, the subject has only received sporadic attention since then. With the advancement of imaging technology, non‐invasive methods can be used to explore the degree of respiratory system development in embryos of different ages.

**Results:**

Micro‐computed tomography (micro‐CT) was used to reconstruct three‐dimensional extrapulmonary airways and pulmonary structures and to analyze lung development in five species of lizards (two species of Teiidae, one Anguidae, one Iguanidae, and one Tropiduridae) and one species of snake (Lamprophiidae). Results indicate that pulmonary parenchyma development was undetectable in the earliest embryonic stages, likely due to technical or developmental limitations. In later stages, structures such as faveolar parenchyma and intrapulmonary subdivisions were clearly observable, resembling the morphology seen in adult animals.

**Conclusions:**

These findings provide valuable insights into the viability of micro‐CT scans to investigate embryonic respiratory systems, as well as into the evolution and development of the respiratory system in Squamata.

## Key Findings

- μCT‐scanning of reptilian embryos provides valuable insight into the development of the respiratory system.
- Detailed assessments of macroscopic pulmonary structures can be performed while maintaining the physical integrity of the specimen.
- Late stage embryos pulmonary structure closely resembles the one seen in adult animal.

## 1 INTRODUCTION

The origin and development of amniote lungs exhibit unique characteristics that reflect the complexity and diversity of this respiratory system. Most amniote taxa show a unique Bauplan regarding lung type, such as the bronchioalveolar lung of mammals, the multichambered lungs of chelonians and crocodilians, or the avian respiratory system with its air sacs and parabronchial lungs.^1^ Within lepidosaurian reptiles, on the other hand, lungs can be shaped as simple sacs (= unicameral or single‐chambered lungs), possessing only few internal subdivisions (= paucicameral or transitional lungs), as well as showing several chambers (= multicameral lungs).^2^

Though the morphological diversity of the adult reptilian respiratory system has been the subject of many studies,^2^, ^3^, ^4^, ^5^, ^6^, ^7^, ^8^, ^9^, ^10^, ^11^, ^12^, ^13^, ^14^ investigations regarding the embryological development of reptilian lungs remain limited, particularly in Squamata. The first such study was carried out by Rathke,^15^ describing in detail lung development in the common grass snake *Natrix natrix* (Colubridae). Rathke^15^ noted that the respiratory system was not yet formed in an embryo of 3.8 mm in length, but in an embryo of about 20 mm in length the trachea and two lung *anlagen* were present, the left one being shorter than the right one. In embryos of about 45 mm in length, the trachea, which does not yet contain cartilaginous rings, and the right lung increase in length, while the left lung remains very small. An embryo of 120–135 mm showed a trachea reaching caudally to the heart, still containing cartilaginous rings in formation, and an elongated right lung with faveolar parenchyma in the cranial part and no respiratory parenchyma in the caudal part. A rudimentary left lung, whose lumen has been almost entirely reduced at the end of development, was present, serving as an anatomical landmark between the trachea and the right lung, since the caudal part of the trachea is wide and forms a direct continuation with the right lung.^15^

While Rathke^15^ provided detailed descriptions for every system within the embryos, not specifically highlighting the respiratory system, subsequent embryological studies mostly looked at non‐avian reptiles with the intention to understand the evolution of the complex bronchial patterns seen in mammalian and avian respiratory systems, investigating the formation of intrapulmonary subdivisions in the other vertebrate classes. Two opposing hypotheses were explored, one where, beginning from a central tube, buds will form that eventually increase in size to result in pulmonary niches/lobes/chambers (centrifugal growth; seen in mammals and birds), while the other hypothesis suggested a centripetal growth of subdivisions starting from the surface of a pulmonary sac, increasing in size and number by growing inwards, thereby forming intrapulmonary divisions. The various arguments raised by the different researchers studying respiratory system development over time for one or the other hypothesis should be reviewed in detail elsewhere, but the matter has been decisively put to an end by Broman,^16^, ^17^ confirming that the centripetal hypothesis lacks any embryological support. Interestingly, Moser^18^ already provided strong evidence in favor of intrapulmonary subdivisions being formed by budding off from the central lumen, but her findings were largely ignored, despite studying embryonic lung development in several squamates, as well as in amphibians, the tuatara *Sphenodon*, a turtle, a crocodile, and the chicken. [Correction added on 25 July 2025, after first online publication: The word “lizard” has been removed from the preceding sentence in this version.]

Other important studies include Schmalhausen,^19^ who revisited the embryological development of *N. natrix*, focusing on the differential growth of left and right lungs, analyzing lung length and parenchyma, showing that in the oldest embryo (8 cm long) the left lung was 0.54 mm and the right lung 20 mm in length, while in the adult the left lung measured 2–4 mm and the right lung 20–30 cm in length. Similar data regarding lung length were previously presented by Butler,^20^ suggesting that in embryos of 5.2 mm diameter, the left lung reaches its greatest length, remaining mostly unaltered in the older embryos he studied. Butler,^20^ Broman,^21^ and Hochstetter^22^ while studying mainly the visceral topology and mesenteric recesses, also commented on the degree of pulmonary development in different embryonic stages, without elaborating on pulmonary parenchyma. Butler^20^ describes some older ophidian embryos where lungs were already present, and also comments on the presence/absence of the left lung in several species of snakes. Broman^21^ studied several species of squamates, providing information regarding the presence or absence of lungs in different stages. Heilmann^23^ mostly reviews the previous literature focusing on the manner of intrapulmonary division during development and presents some data for embryological lungs of *Lacerta agilis*, while Peter^24^ commented only superficially on the respiratory system in his embryological staging tables of *L. agilis*. Schmidt^25^ and Böker^26^ investigated the formation of the trachea and glottis, not investigating the lungs themselves, while Broman^27^ examined embryos of the side‐striped chameleon *Trioceros bitaeniatus* (Chamaeleonidae) and observed that at 5 mm the respiratory system is composed of trachea, extrapulmonary bronchi, and lung *anlagen*, the latter with initial dorsal and ventral buds which eventually will form chambers in the paucicameral lung. In 6 mm embryos, faveolar respiratory parenchyma was visible, especially in the cranial part of the lungs.

In recent years, several species of snakes had their asymmetric lung development investigated,^28^ while the involvement of Sox genes in the morphogenesis of embryonic corn snake *Pantherophis guttatus* lungs was studied by van Soldt et al.^29^ Palmer et al.^30^ looked at the role of intrapulmonary smooth muscles during the formation of the lung in the brown anole *Anolis sagrei* (Dactyloidae). Lambertz et al.^31^ found that the early branching stages of lung development in the Madagascar ground gecko *Paroedura picta* (Gekkonidae) resembled those of multicameral lungs of other amniotes. They hypothesized that the pulmonary structures observed in Lepidosauria might be the result of secondary simplification during evolution, since a multicameral condition remains visible in certain developmental stages of *P. picta*, despite this species possessing unicameral lungs as adults.

Our summary (Table 1) of the available descriptions for embryonic squamate lungs clearly shows that only a small number of species have been studied thoroughly throughout their entire development, and for some species, only isolated embryonic stages are available, while the vast majority of snakes and lizards lack any description regarding the formation of the respiratory system. In recent years, studies investigating the molecular and genetic framework involved in the formation of the respiratory system have become more frequent,^32^, ^33^ investigating branching patterns,^34^, ^35^ or the homology of vertebrate lungs,^36^ highlighting the renewed importance regarding the embryological development of the vertebrate pulmonary system, especially within the diverse lung morphologies seen among squamates.

**TABLE 1:** List of lepidosaurian species whose respiratory system has been studied in one or more embryonic stages. Adult lung type (U—unicameral, P—paucicameral, M—multicameral), when available.

Family	Species	Adult lung type	Embryonic stage	References
Rhynchocephalia				
Sphenodontidae	*Sphenodon punctatus*	U	N.A.	^18^
Sauria				
Agamidae	*Calotes* sp.	U	16 embryos from 11.0 to 40.0 mm	^25^
Anguidae	*Anguis fragilis*	U	4.0, 5.0, 5.25, 7.0 mm	^18^
			4.2 mm	^37^
			10 embryos from 3.5 to 8.2 mm diameter	^21^
			1.2 mm head length	^22^
	*Elgaria multicarinata*	U	12, 29, 40 dpo	This study
Chamaeleonidae	*Trioceros bitaeniatus*	P	12 embryos from 4.5 to 15.5 mm	^27^
Dactyloidae	*Anolis sagrei*	U	Embryonic Days 5–7	^30^
Gekkonidae	*Gehyra oceanica*	‐	2.8, 3.2 mm	^18^
	*Paroedura picta*	U	10–50 dpo	^31^
	*Gekko* sp.	U	15 embryos from 6.1 to 35.0 mm	^25^
Iguanidae	*Iguana iguana*	P	22, 36 dpo	This study
Lacertidae	*Lacerta agilis*	U	5 embryos from 2.0 to 6.5 mm	^21^
			N.A.	^22^
			127 embryos from conception up to 12.0 mm	^24^
			N.A.	^26^
	*Lacerta viridis*	U	N.A.	^22^
	*Podarcis muralis*	U	2.0, 3.5 mm	^18^
	*Zootoca vivipara*	U	4.5, 5.5 mm	^18^
			3.8 mm head length	^23^
Phyllodactylidae	*Tarentola* sp.		3.0, 4.4, 6.0, 8.5, 9.0 mm	^37^
	*Tarentola mauritanica*	U	7 embryos from 3.0 to 9.0 mm	^21^
Phrynosomatidae	*Sceloporus undulatus*	‐	6.0 mm	^21^
Scincidae	*Mabuya*	U	19 embryos from 7.4 to 43.0 mm	^25^
Teiidae	*Aspidoscelis sexlineata*	U	10 embryos from 5.0 to 27 mm	^21^
	*Aspidoscelis uniparens*	U	25, 44, 53 dpo	This study
	*Cnemidophorus* sp.	U	8.2, 20.2 mm	^37^
	*Salvator merianae*	U	8.2 mm	^21^
			18, 30 dpo	This study
Tropiduridae	*Tropidurus catalanensis*	‐	Stages 34–35 (18–23 dpo^38^); Stage 39 (39–50 dpo^38^)	This study
Serpentes				
Colubridae	*Elaphe quatuorlineata*	‐	110, 115 mm	^20^
	*Elaphe obsoleta*	‐	22 dpo	^28^
	*Erythrolamprus aesculapii*	U	N.A.	^22^
	*Natrix natrix*	U	~ 3.7, 22.6, 45.2, 135, 215 mm	^15^
			N.A.	^19^
			N.A.	^20^
			2.8, 3.3, 5.2, 10.0 mm diameter	^21^
			N.A.	^22^
	*Hierophis gemonensis*	U	N.A.	^20^
	*Pantherophis guttatus*	U	2–51 dpo	^28^, ^29^
Lamprophiidae	*Boaedon capensis*	‐	13, 23, 41 dpo	This study
Pythonidae	*Python curtus breitensteini*	U	2, 5, 10, 14 dpo	^29^
Viperidae	*Calloselasma rhodostoma*	U	3, 20 dpo	^29^
	*Causus rhombeatus*	U	5, 8 dpo	^29^
	*Vipera aspis*	U	N.A.	^20^

Abbreviations: dpo, days post‐oviposition; N.A., not available.

Microtomographic techniques have been well established to investigate the respiratory system of vertebrates.^39^, ^40^, ^41^ Among squamates, Cieri et al.^42^ investigated the green iguana (*Iguana iguana*) and Schachner et al.^39^ the savannah monitor *Varanus exanthematicus* regarding intrapulmonary airflow patterns. Low resolution scans were performed on the bearded dragon *Pogona vitticeps*,^43^ the common collared lizard *Crotaphytus collaris*, and the long‐nosed leopard lizard *Gambelia wislizenii* ^44^ to investigate functional aspects of the respiratory system, while computed tomography is also a valuable diagnostic tool in veterinary medicine.^45^, ^46^, ^47^

Studies using micro‐CT‐scanning to investigate the degree of respiratory system development in squamate embryos are lacking up to now. Therefore, the present investigation intends to evaluate the viability of micro‐CT scans of embryos of the desert grassland whiptail lizard *Aspidoscelis uniparens* and *Salvator merianae* (Teiidae), the southern alligator lizard *Elgaria multicarinata* (Anguidae), *I. iguana* (Iguanidae), the neotropical ground lizard *Tropidurus catalanensis* (Tropiduridae), and the Cape house snake *Boaedon capensis* (Lamprophiidae). The embryonic series obtained from scientific collections will be used to reconstruct the degree of respiratory system development at different embryonic stages, with special attention to the trachea, any extrapulmonary bronchi, the lungs, and the differentiation of pulmonary parenchyma.

## 2 RESULTS

### 2.1 Teiidae

**FIGURE 1:**
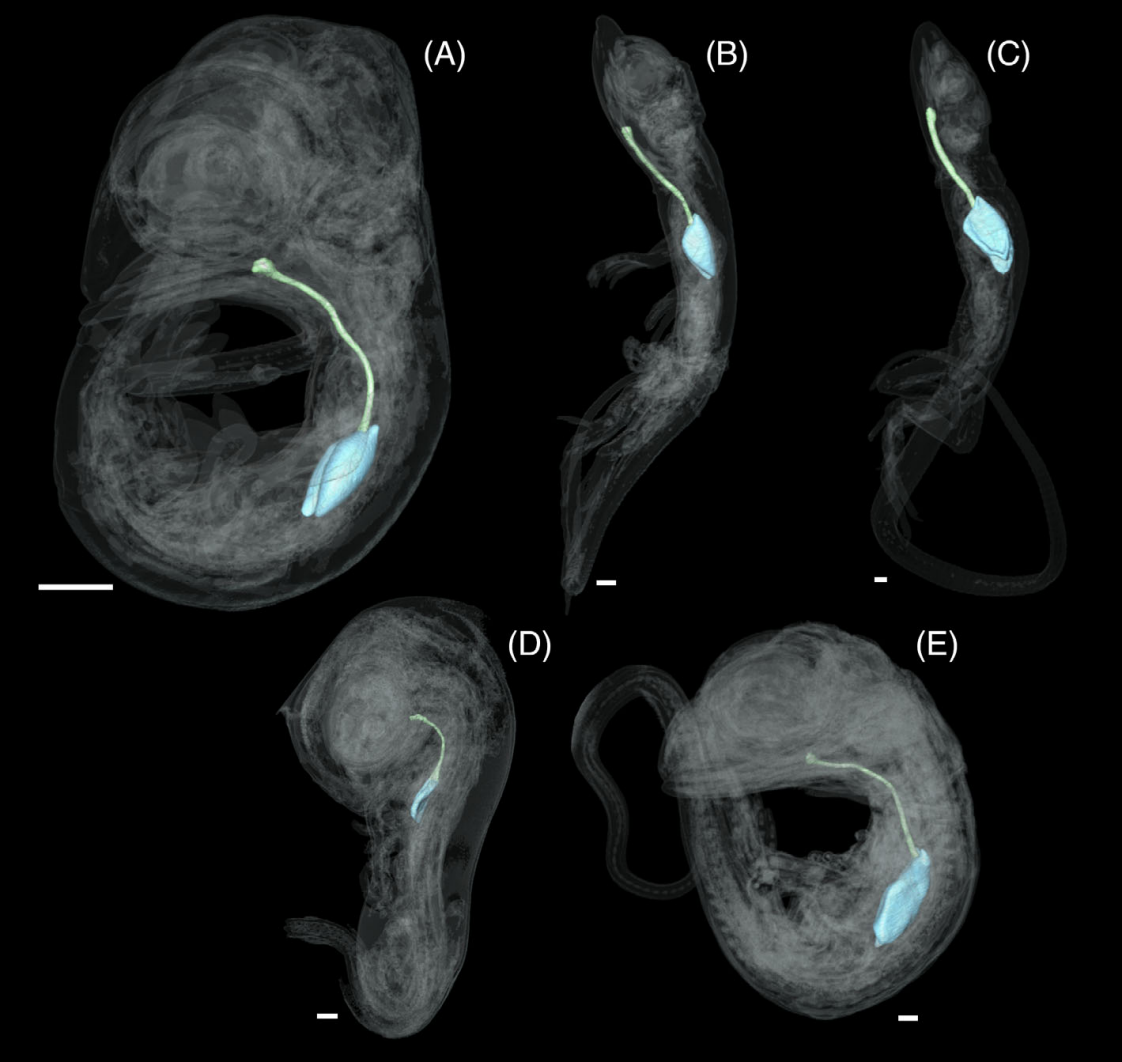
Images showing the entire embryos and the localization of the respiratory system within each Teiidae specimen. (A) *Aspidoscelis uniparens* (25 days post‐oviposition [dpo]). Voxel size 8.88 × 8.88 × 8.88 μm. (B) *A. uniparens* (44 dpo). Voxel size 17.77 × 17.77 × 17.77 μm. (C) *A. uniparens* (53 dpo). Voxel size 19.99 × 19.99 × 19.99 μm. (D) *Salvator merianae* (18 dpo). Voxel size 18.02 × 18.02 × 18.02 μm. (E) *S. merianae* (30 dpo). Voxel size 18.02 × 18.02 × 18.02 μm. The tracheal mold is in white, and the lungs are in blue. Scale bars 1 mm.

**FIGURE 2:**
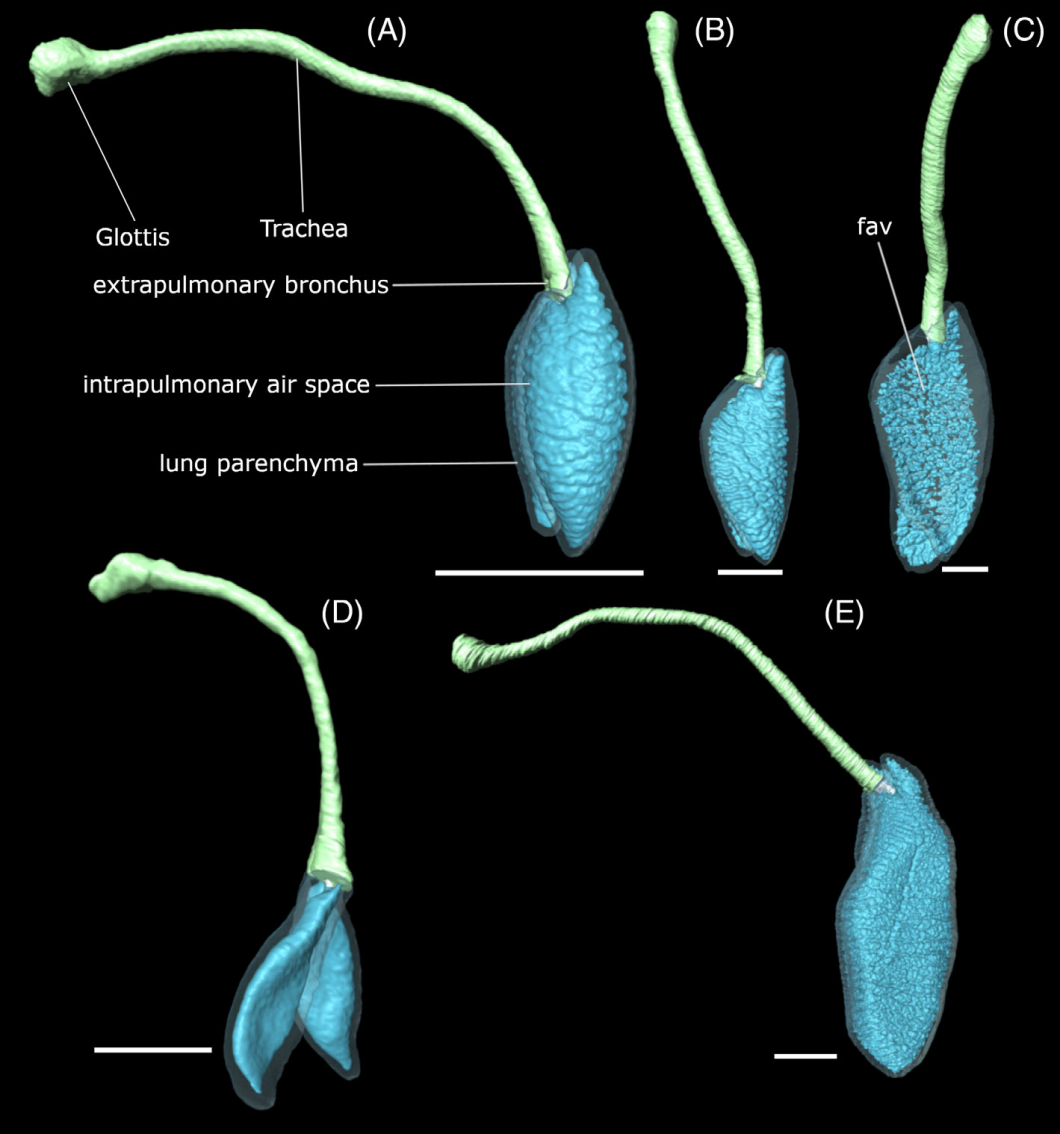
Reconstructions of embryonic Teiidae respiratory systems. (A) Left lateral view of the respiratory system of *Aspidoscelis uniparens* (25 days post‐oviposition [dpo]). Voxel size 8.88 × 8.88 × 8.88 μm. (B) Left lateral view of the respiratory system of *A. uniparens* (44 dpo). Voxel size 17.77 × 17.77 × 17.77 μm. (C) Left lateral view of the respiratory system of *A. uniparens* (53 dpo). Voxel size 19.99 × 19.99 × 19.99 μm. (D) Left lateral view of the respiratory system of *Salvator merianae* (18 dpo). Voxel size 18.02 × 18.02 × 18.02 μm. (E) Left lateral view of the respiratory system of *S. merianae* (30 dpo). Voxel size 18.02 × 18.02 × 18.02 μm. The tracheal mold is in white. The intrapulmonary air spaces are represented in blue and the translucent outline of the lungs (= pulmonary parenchyma) is shown in gray. fav = faveolar epithelium. Scale bars 1 mm.

Figure 1 provides an overview of the position of the respiratory system within each embryo analyzed, as well as the proportions of extrapulmonary airways and lungs. At 25 dpo (days post‐oviposition), the *A. uniparens* embryo displayed a trachea and extrapulmonary bronchi connected to two well‐developed unicameral lungs with evident parenchyma (Figures 1A and 2A). The intermediate stage (44 dpo) revealed similar characteristics (Figure 2B). In the most advanced stage (53 dpo), the parenchyma appeared highly subdivided (Figure 2C). In the youngest stage analyzed (18 dpo), *S. merianae* exhibited two sac‐shaped unicameral lungs without dense respiratory parenchyma, connected to the trachea via small extrapulmonary bronchi (Figure 2D). In the more advanced specimen (30 dpo), the lungs showed a similar structure, but with discernible, well‐subdivided parenchyma (Figure 2E).

### 2.2 Iguanidae

**FIGURE 3:**
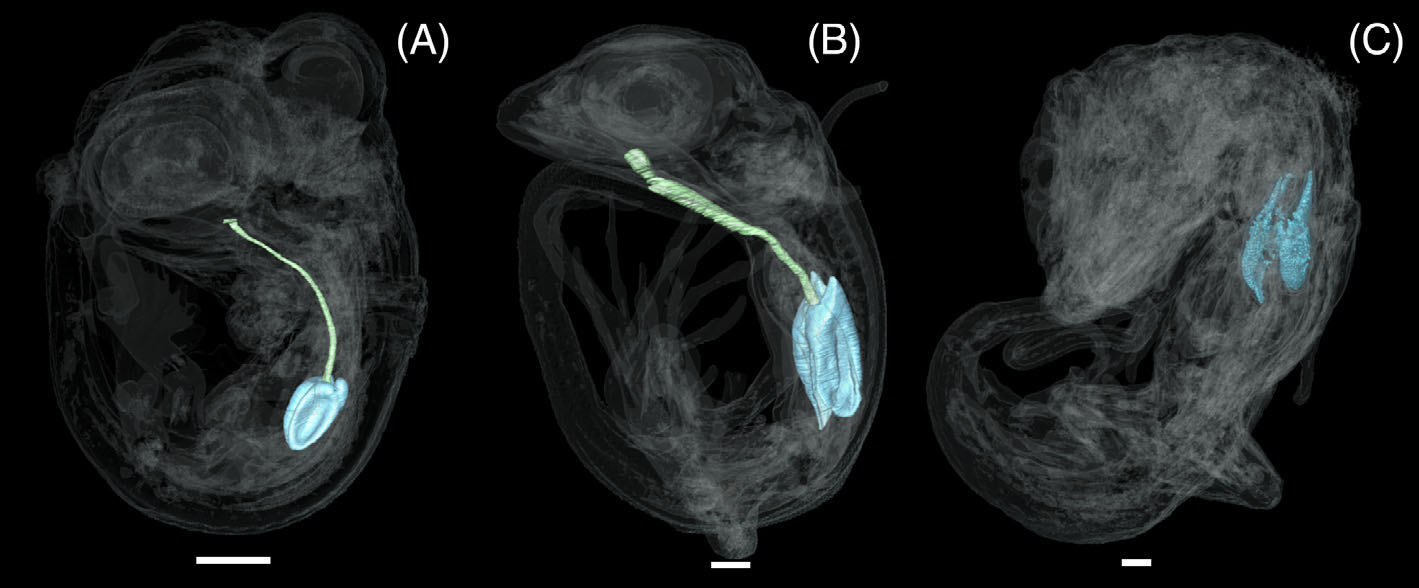
Images showing the entire embryos and the localization of the respiratory system within each Iguania embryo. (A) *Tropidurus catalanensis* (embryonic Stages 34–35 corresponding to 18–23 days post‐oviposition [dpo]^38^). Voxel size 8.70 × 8.70 × 8.70 μm. (B) *T. catalanensis* (Stage 39, corresponding to 39–50 dpo^38^). Voxel size 15.33 × 15.33 × 15.33 μm. (C) *Iguana iguana* (36 dpo; no extrapulmonary airway was discernable in this embryo due to limited image contrast and noise). Voxel size 14.56 × 14.56 × 14.56 μm. The tracheal mold is in white, and the lungs are in blue. Scale bars 1 mm.

**FIGURE 4:**
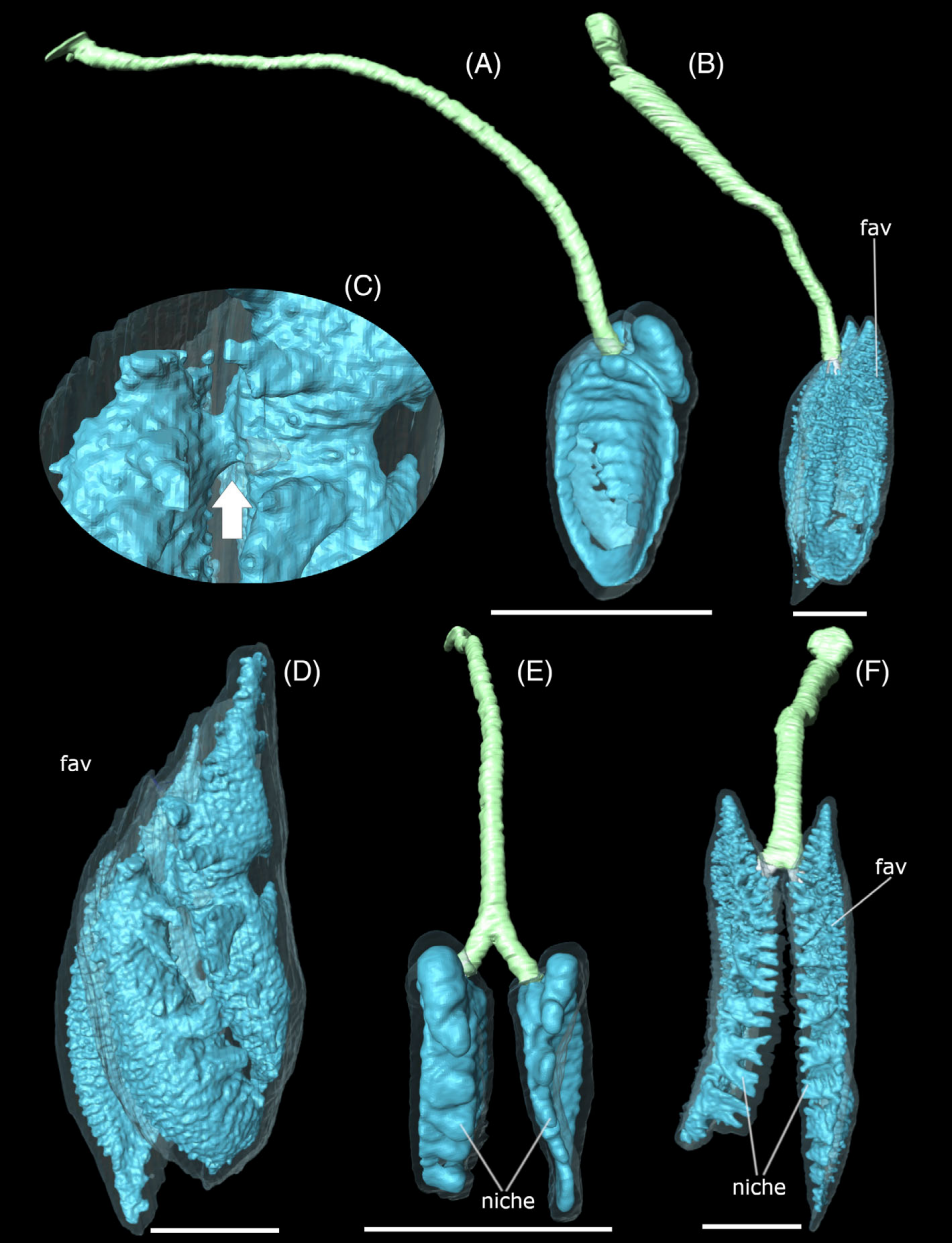
Reconstructions of embryonic Tropiduridae and Iguanidae respiratory systems. (A) Left lateral view of the respiratory system of *Tropidurus catalanensis* (embryonic Stages 34–35 corresponding to 18–23 days post‐oviposition [dpo]^38^). Voxel size 8.70 × 8.70 × 8.70 μm. (B) Left lateral view of the respiratory system of *T. catalanensis* (embryonic Stage 39, corresponding to 39–50 dpo^38^). Voxel size 15.33 × 15.33 × 15.33 μm. (C) Left lateral view of a lung of *Iguana iguana* (36 dpo), focusing on the connection between the cranial and caudal pulmonary chambers (→). Voxel size 14.56 × 14.56 × 14.56 μm. (D) Left lateral view of the lungs of *I. iguana* (36 dpo). Voxel size 14.56 × 14.56 × 14.56 μm. (E) Dorsal view of the respiratory system of *T. catalanensis* (embryonic Stages 34–35 corresponding to 18–23 dpo^38^), highlighting the initial dorsal niches. Voxel size 8.70 × 8.70 × 8.70 μm. (F) Dorsal view of the respiratory system of *T. catalanensis* (embryonic Stage 39, corresponding to 39–50 dpo^38^), highlighting the dorsal niches. Voxel size 15.33 × 15.33 × 15.33 μm. The tracheal mold is in white. The intrapulmonary air spaces are represented in blue and the translucent outline of the lungs (= pulmonary parenchyma) is shown in gray. fav = faveolar epithelium. Scale bars 1 mm.

The *I. iguana* embryo where the respiratory systems could be observed is presented in Figure 3C, while in the earliest developmental phase analyzed (22 dpo) no lung development could be observed. By 36 dpo, two paucicameral lungs were identified, subdivided into a cranial and a caudal chamber with well‐developed epithelial linings (Figure 4C,D). The segmentation of the trachea and bronchi was not feasible at this stage due to limited image contrast and noise.

### 2.3 Tropiduridae

Figure 3A,B shows the respiratory systems located within each *Tropidurus* specimen. In the youngest stage (embryonic Stages 34–35 corresponding to 18–23 dpo), *T. catalanensis* embryos had two well‐developed lungs connected to the trachea via extrapulmonary bronchi (Figure 4A). The dorsal region of the lungs featured rudimentary niches; however, at this stage, the type of respiratory parenchyma could not be discerned (Figure 4E). In the more advanced stage (embryonic Stages 39, corresponding to 39–50 dpo), the parenchyma exhibited more extensive and elaborate development, and niches were clearly discernible in the dorsalmost part of each lung (Figure 4B,F).

### 2.4 Anguidae

**FIGURE 5:**
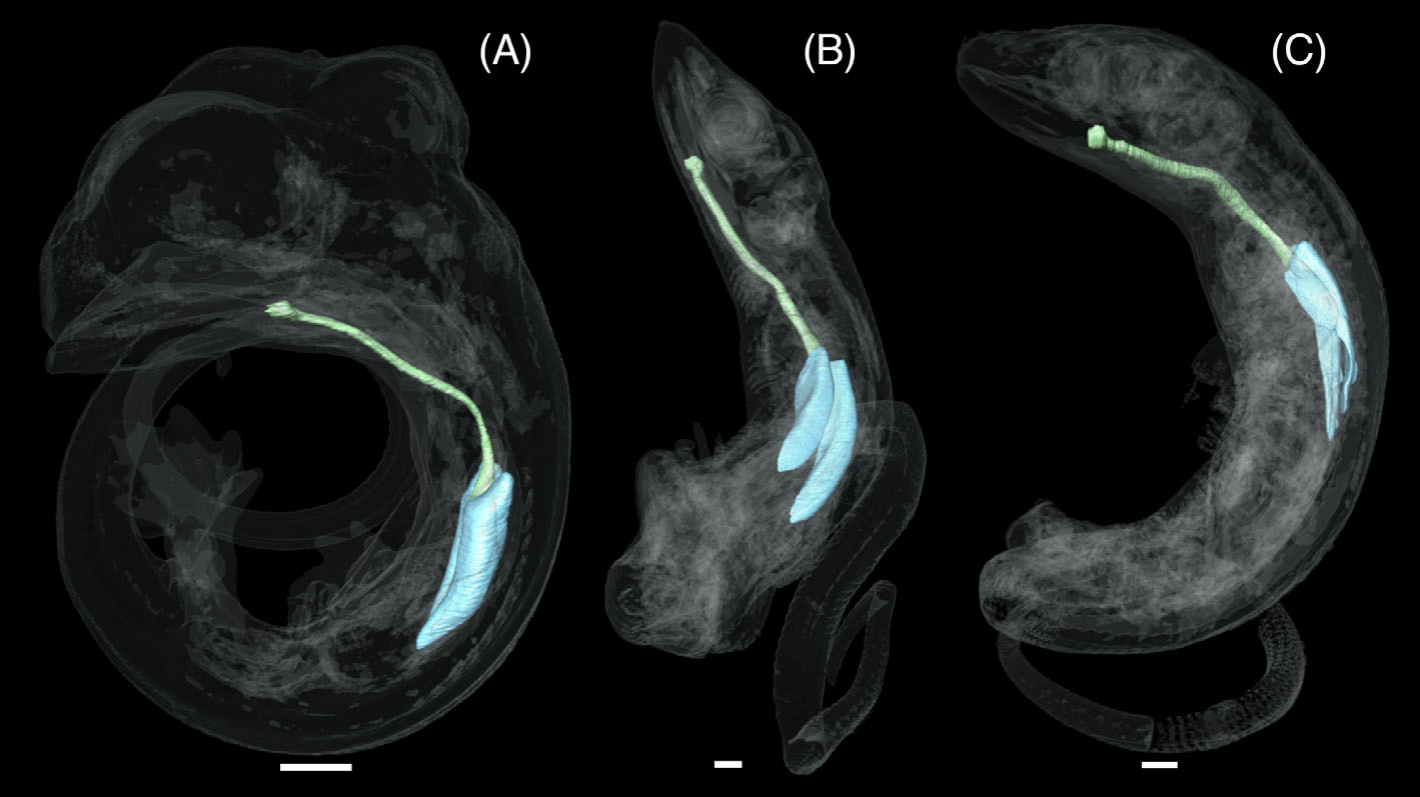
Images showing the entire embryos and the localization of the respiratory system within Anguimorpha embryos. *Elgaria multicarinata* (A) 12 days post‐oviposition [dpo]. Voxel size 12.44 × 12.44 × 12.44 μm. (B) 29 dpo. Voxel size 15.55 × 15.55 × 15.55 μm. (C) 40 dpo. Voxel size 15.55 × 15.55 × 15.55 μm. The tracheal mold is in white, and the lungs are in blue. Scale bars 1 mm.

**FIGURE 6:**
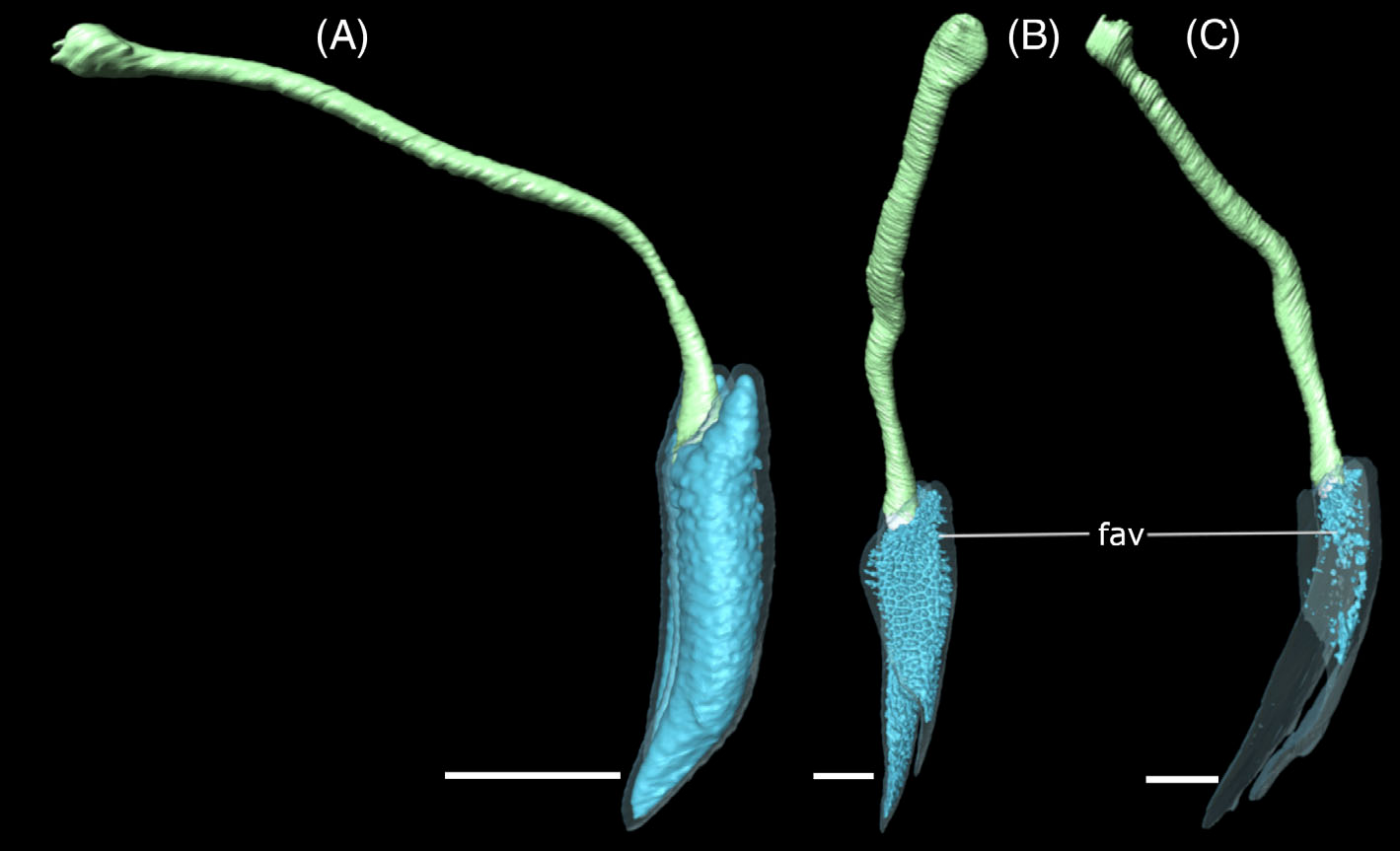
Reconstruction of embryonic Anguimorpha respiratory systems. (A) Left lateral view of the respiratory system of *Elgaria multicarinata* (12 dpo). Voxel size 12.44 × 12.44 × 12.44 μm. (B) Left lateral view of the respiratory system of *E. multicarinata* (29 dpo). Voxel size 15.55 × 15.55 × 15.55 μm. (C) Left lateral view of the respiratory system of *E. multicarinata* (40 dpo). Voxel size 15.55 × 15.55 × 15.55 μm. The tracheal mold is in white. The intrapulmonary air spaces are represented in blue and the translucent outline of the lungs (= pulmonary parenchyma) is shown in gray. fav = faveolar epithelium. Scale bars 1 mm.

The three investigated Anguimorpha embryos are presented in Figure 5. In *E. multicarinata*, the lungs were unicameral across all stages analyzed. At 12 dpo, the lungs were similar in size, with shallow parenchyma (Figure 6A). By 29 dpo, the parenchyma became densely subdivided, extending throughout the lungs, and size asymmetry was evident (Figure 6B). At 40 dpo, parenchyma was primarily concentrated in the cranial region, while the caudal region lacked gas exchange tissue (Figure 6C). However, it is important to note that the lungs appear to be collapsed, which may affect the accuracy of the results regarding the true extent of parenchymal development.

### 2.5 Lamprophiidae

**FIGURE 7:**
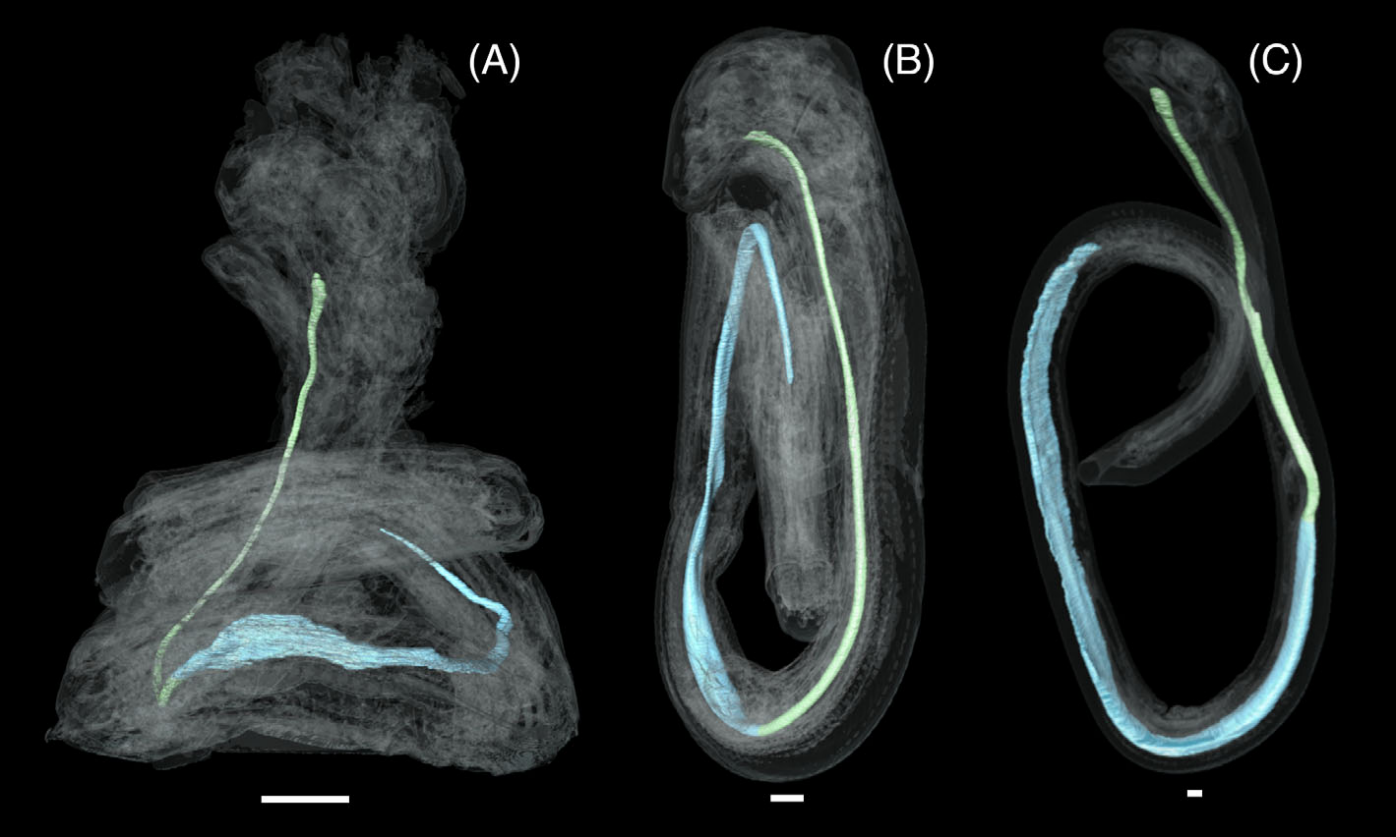
Images showing the entire embryos and the localization of the respiratory system within embryonic specimens of Serpentes. *Boaedon capensis* (A) 13 days post‐oviposition [dpo]. Voxel size 12.44 × 12.44 × 12.44 μm. (B) 23 dpo. Voxel size 15.55 × 15.55 × 15.55 μm. (C) 41 dpo. Voxel size 28.89 × 28.89 × 28.89 μm. The tracheal mold is in white, and the lung is in blue. Scale bars 1 mm.

**FIGURE 8:**
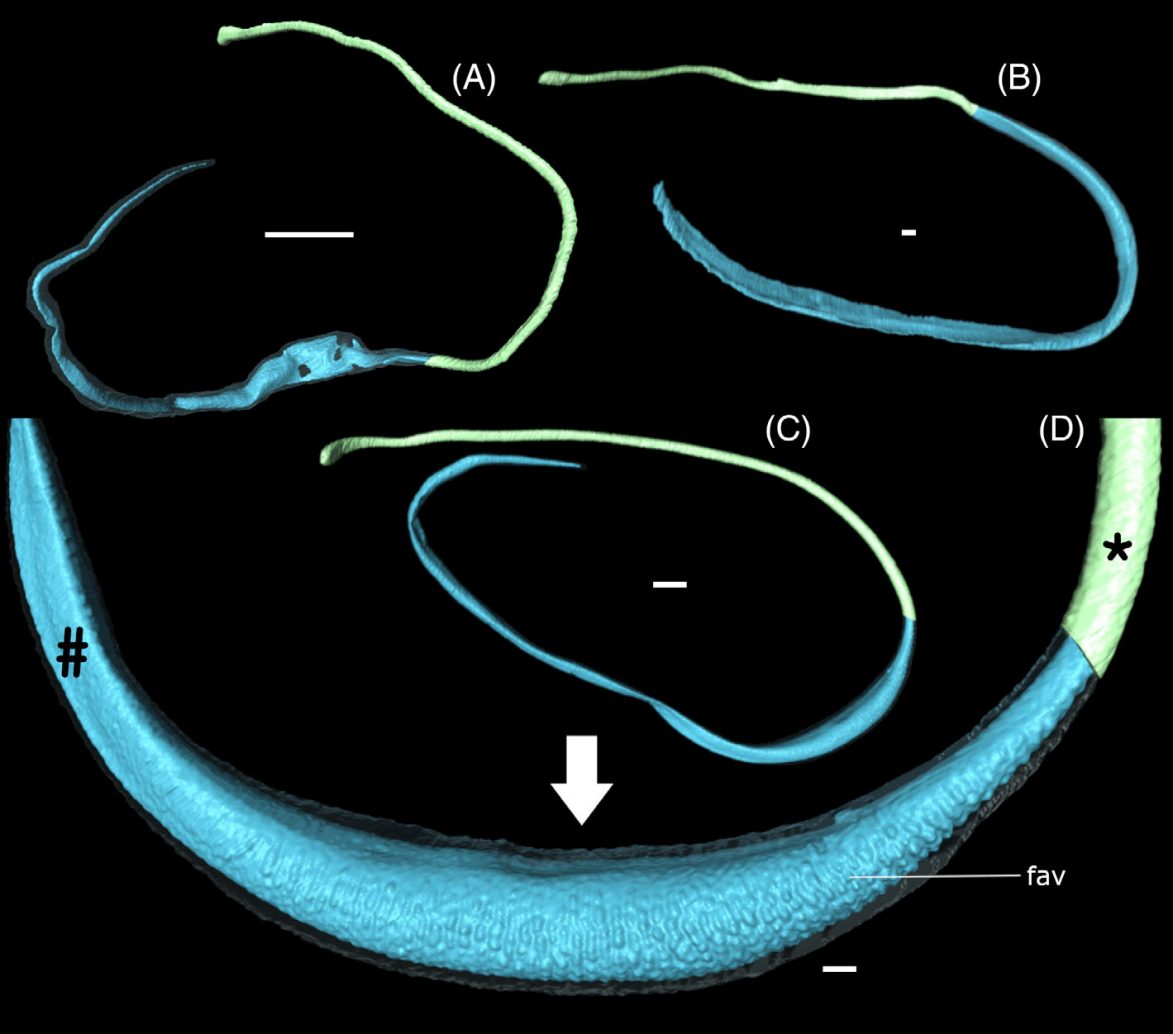
Reconstruction of embryonic Serpentes respiratory systems. (A) Left lateral view of the respiratory system of *Boaedon capensis* (13 days post‐oviposition [dpo]). Voxel size 12.44 × 12.44 × 12.44 μm. (B) Left lateral view of the respiratory system of *B. capensis* (41 dpo). Voxel size 28.89 × 28.89 × 28.89 μm. (C) Left lateral view of the respiratory system of *B. capensis* (23 dpo). Voxel size 15.55 × 15.55 × 15.55 μm. (D) Left lateral view of the lung of *B. capensis* (23 dpo) focusing on the parenchyma (→), air sac (#), and trachea (*). Voxel size 15.55 × 15.55 × 15.55 μm. The tracheal mold is in white. The intrapulmonary air spaces are represented in blue and the translucent outline of the lungs (= pulmonary parenchyma) is shown in gray. fav = faveolar epithelium. Scale bars 1 mm.

The scans from the embryonic specimens of the Serpentes species are given in Figure 7. *B. capensis* exhibited a long trachea and a single functional right lung, with the left lung absent. At 13 dpo, no visible parenchyma was present (Figure 8A). The intermediate stage (23 dpo) revealed parenchyma in the cranial portion of the lung, while the caudal portion remained smooth and devoid of respiratory tissue (Figure 8C,D). At 41 dpo, the lungs maintained a similar structure to the youngest stage, lacking subdivided parenchyma (Figure 8B).

## 3 DISCUSSION

Following the respiratory pharynx hypothesis,^1^ the lungs of sarcopterygians, including crossopterygians, dipnoans, and tetrapods, are homologous, originating from the ventral region of the caudal pharynx and sharing the same arterial supply. In lungfish and tetrapods, the lungs develop from the caudal branchial pouches and receive deoxygenated blood from the heart through the pulmonary arteries, which are derived from the sixth branchial arch. While the lungs of lungfish and amphibians exhibit a similar structure containing a single epithelial cell type in the gas exchange regions, the lungs of amniotes are characterized by two epithelial types, type I cells forming the gas exchange surface and surfactant‐producing type II cells.^1^ In a recent study, Li et al.,^36^ found conserved gene expression patterns related to vertebrate lung development. Embryological studies in amniotes have demonstrated that the lung rudiment begins as a laryngotracheal groove, which is unpaired, tubular, and serves as the precursor for the bronchi and airways.^32^, ^33^, ^34^ In compartmentalized reptilian lungs, which lack intrapulmonary bronchi, the chamber walls develop before the gas exchange tissue. These studies suggest that the compartmentalized structure and airways are ancestral features of the lung, providing further evidence for the hypothesis of a multicameral lung at the origin of amniotes.^48^

The two stages of *S. merianae* analyzed exhibit well‐developed lungs with uniformly distributed respiratory parenchyma. As described in the literature, the adults of this species have a unicameral lung, with a central air‐filled cavity extending radially into the parenchyma.^49^ The younger specimen did not exhibit the well‐developed respiratory parenchyma observed in adults of this species but showed a proportionally longer trachea. The faveolar parenchyma was homogeneously dispersed throughout the organ.^50^, ^51^

The analyzed stages of *A. uniparens* exhibit similar characteristics, with the more advanced stages (Figure 2B,C) displaying parenchyma that can be characterized as faveolar due to its extreme subdivision. No literature data were found regarding the pulmonary characteristics of adults of this species. The species belongs to the Teiidae family, whose members are characterized by unicameral lungs with faveolar epithelium and homogeneous distribution,^50^ consistent with the described embryonic pulmonary anatomy. In the Lacertidae, a family closely related to Teiidae,^52^ Moser^18^ described faveolar epithelium in an embryo of *Zootoca vivipara* of 5.5 mm length, suggesting that this kind of epithelium is commonly developed in Lacertoidea embryos, as seen in adults.^50^

The absence of lung evidence in the younger specimen of *I. iguana* may be attributed either to the lack of lung formation at this stage or to limitations in the employed contrast method, which may have inadequately stained the sample, hindering the visualization of the structure. The more advanced stage exhibited two paucicameral lungs with well‐developed epithelium. At this stage, pulmonary characteristics resembling those found in adults of this species were observed: two chambers separated by a longitudinal septum, with the cranio‐medial chamber composed of faveolar epithelium and the caudal chamber featuring heterogeneous epithelium, including faveolar epithelium in the cranial region and trabecular epithelium in the caudal region.^42^, ^53^, ^54^ The epithelial differentiation in the chambers is associated with greater specialization for gas exchange in the cranial chamber and adaptation for ventilatory function in the caudal chamber.^54^ In this species, the right lung is substantially larger than the left.^7^, ^42^, ^53^

In the stages of *T. catalanensis*, two lungs were observed, with internal anatomy distinct from the previously described species. The dorsal region of the lungs displayed well‐developed niches, similar to those described in other Iguania species.^2^ The parenchyma of the younger specimen showed no apparent subdivision, making it difficult to identify the parenchyma type. In the older specimen, the parenchyma was more deeply developed and appeared to be faveolar, and apparently showed several dorsal niches in both lungs (Figure 4F). No descriptions of the pulmonary anatomy of this species were found in the literature. Although Iguania species are generally described as having paucicameral lungs,^2^ the clade currently comprises 14 distinct families, only half of which have species with descriptions of their pulmonary anatomy. The embryos of the investigated Tropiduridae species showed no evidence of a paucicameral lung structure, more of a homogenous lung with dorsal niches.

In *E. multicarinata*, the lungs in the initial stage were of approximately equal size, with shallow parenchyma suggesting being of the trabecular kind. In the intermediate stage, the parenchyma can clearly be characterized as faveolar, extending homogeneously throughout each lung. In the advanced stage, the parenchyma seemed to be more concentrated cranially, but the compression of the embryonic lung impedes further considerations. No data on the adult lungs of this species were found in the literature. The Anguidae family includes species with paucicameral lungs with edicular epithelium or unicameral lungs.^2^ At the developmental stages investigated, the lungs appear to be unicameral. Any pulmonary asymmetry observed in *E. multicarinata* embryos must be taken with caution, since only one animal per embryonic stage was investigated. Furthermore, depending on the anguid species studied, lungs may show left lungs being consistently smaller, as seen in the common slow worm *Anguis fragilis*, or either the left or the right lung might be smaller than the other, as seen in the sheltopusik, *Pseudopus apodus*.^55^

The embryos of *B. capensis* exhibit pulmonary structures typically described for snakes: a long trachea, an elongated right lung, with the cranial region composed of specialized gas exchange tissue, and a caudal region devoid of parenchyma, known as the air sac.^2^, ^14^ In the youngest analyzed stage, no significant parenchymal development was observed, which may indicate that tissue differentiation had not yet begun, while the intermediate stage suggests faveolar parenchyma in the cranialmost region of the lungs (Figure 8D). The more advanced stage showed no clear respiratory parenchyma; however, this absence could be attributed to technical challenges in obtaining the images, as vibrations compromised the precise segmentation of visceral details. No data were found for adults of this species, but for the genus *Boaedon*, the literature indicates the absence of a tracheal lung and faveolar parenchyma,^14^ consistent with the observed embryonic anatomy.

Lambertz et al.^31^ proposed that multicameralization represents an ancestral trait in amniotes, with its simplification in extant species likely having been driven by body miniaturization during the evolution of squamate ancestors, a strategy that improves respiratory efficiency in smaller reptiles.^49^ During the ontogeny of *P. picta* (Gekkota), multicameralization has been observed as a sequence of budding events that form dorsal and ventral chambers, with the anterior elements progressively increasing in size and width. This process has also been described for the chameleon lung by Broman.^27^

Although Lambertz et al.^31^ did not specify the embryonic ages analyzed, visual evidence from their study suggests that the multicameral branching phase occurs between approximately 10 and 20 dpo, as indicated by comparisons with the embryonic timelines established by Noro et al.^56^ Among the embryos examined in the present study, only *A. uniparens* falls outside this developmental range. Despite this overlap, none of the younger embryos analyzed here exhibit evidence of multicameral differentiation, as indicated by dorsal and ventral subdivisions of the respiratory parenchyma. However, Lambertz et al.^31^ also studied the pulmonary circulatory system to demonstrate intrapulmonary separation into multiple chambers, a parameter not available in the current study due to the limitations of the imaging methods used.

Consequently, while no direct evidence of multicameralization was observed in the species analyzed, this absence cannot be interpreted as definitive proof of its exclusion. Dorsal niches are common among lizards with unicameral lungs,^2^, ^50^ and might be secondary formations, not related to an embryonic multicameral lung as seen in *P. picta*. Observing dorsal and ventral niches within embryonic lungs would provide strong evidence for a multicameral stage in support of Lambertz et al.^31^ hypothesis. Furthermore, lung development may begin at different stages across species, and the branching phase may occur prior to the stages examined by us or could be restricted to *P. picta* or other species of Gekkota. Since no data regarding the pulmonary structure of adult *T. catalanesis* are currently available, it seems worthwhile investigating the extension of niches in the adult lungs, as well as studying the pulmonary circulatory system of embryonic stages of this species using histological techniques.

We used micro‐computed tomography (micro‐CT) to study embryonic lung morphology non‐invasively. Recent advances in micro‐CT technology have provided researchers with a robust tool for documenting, quantifying, and visualizing lung anatomy with high precision and reliability.^40^, ^57^ The application of three‐dimensional segmentation and reconstruction methods allowed for a detailed assessment of macroscopic pulmonary structures while preserving the physical integrity of the specimens. However, micro‐CT imaging has inherent limitations. The technique does not facilitate detailed identification of epithelial cell types, nor pulmonary musculature, nor vasculature. Specimens in this study were not prepared specifically for respiratory system analyses, which may have resulted in noise and compromised the resolution of minute structures. While common species might be reproduced more frequently for scientific purposes and might therefore be available in larger numbers, others are particularly scarce to obtain. For example, *T. catalanensis*, *I. iguana*, and *S. merianae* embryos were obtained from previous studies not related to the study of the respiratory system, and embryos of *E. multicarinata* have been made available for the first time in the present study for scientific research. Future investigations could address these issues by employing fixation protocols tailored toward the respiratory system, which would preserve pulmonary and circulatory structures more effectively and provide additional insights into pulmonary development.

The limitations of this study's imaging approach, coupled with the potential absence of multicameralization in the examined species or the lack of appropriately staged embryos, likely contributed to the absence of significant findings regarding multicameral lung formation. Nevertheless, the analysis of embryonic stages across multiple Squamata species offers valuable insights into the evolutionary and developmental dynamics of the respiratory system, even when only a minimal sample size is available for each developmental stage. Morphological characters observed in embryos, such as intrapulmonary subdivisions, presence of extra‐ or intrapulmonary bronchi, type of respiratory parenchyma, that are also present in adults of the same species, support the validity of non‐meristic morphological observations.

Given the scarcity of studies in this area, the application of micro‐CT demonstrates significant potential for advancing our understanding of lung evolution. As indicated by the compilation given in Table 1, most species studied show unicameral lungs as adults and only two species possess paucicameral lungs. Surprisingly, no lizard species possessing multicameral lungs had any embryonic pulmonary stage investigated. It therefore seems necessary to investigate the embryological development of the respiratory system in species of gila monsters *Heloderma*, monitor lizards *Varanus*, or the earless monitor lizard *Lanthanotus*. These taxa possess multicameral lungs^2^ and the pattern of lung differentiation seen within those lizards should be compared with the patterns seen leading to unicameral or paucicameral lungs.

If future micro‐CT studies identify evidence of multicameralization during specific developmental stages, complementary techniques such as histology should be employed to provide a more detailed assessment of pulmonary structure and differentiation. These combined approaches will be crucial for unraveling the complexities of lung development in squamates and other amniotes. Thus, this study not only enhances our knowledge of pulmonary morphology in Squamata but also establishes a solid foundation for future research that can further explore the relationship between structure, function, and the evolution of the respiratory system in these groups.

## 4 EXPERIMENTAL PROCEDURES

### 4.1 Sample preparation and imaging

Embryos of *I. iguana* (*N* = 2; 22 and 36 dpo), *S. merianae* (*N* = 2; 18 and 30 dpo), and *T. catalanensis* (*N* = 2; embryonic Stages 34–35 corresponding to 18–23 dpo^38^; Stage 39, corresponding to 39–50 dpo^38^) were digitized using micro‐CT at the Biodiversity Documentation Center (Faculdade de Filosofia, Ciências e Letras de Ribeirão Preto‐Universidade de São Paulo ‐ FFCLRP‐USP), employing a GE Phoenix v|tome|xs 240 high‐resolution industrial micro‐CT scanner. The phosphotungstic acid (PTA) contrast method, as described by Lesciotto et al.,^58^ was used to enhance visualization of biological soft tissues. The neotropical embryos used in this study were obtained under federal approval (Instituto Chico Mendes de Conservação da Biodiversidade—SISBIO number 35221) and were deposited at the Coleção Herpetológica de Ribeirão Preto (CHRP, FFCLRP‐USP; *I. iguana*—CHRP 5718 (batch 1381); *S. merianae*—CHRP 5719 (batch 1382); *T. catalanensis*—CHRP 6047).

Embryos preserved in different media were transferred to 1× phosphate‐buffered saline (PBS) for a series of 24‐, 48‐, and 72‐h exchanges and maintained in PBS until the contrast application. They were then immersed in a 3% PTA solution in 1× PBS for 5 days. To ensure tissue stability and prevent dehydration, embryos were encapsulated in clear gelatin within Falcon tubes during the scanning process. After imaging, the gelatin was dissolved in a water bath, and the embryos were returned to their original storage solution.

Embryos of *A. uniparens* (*N* = 3; 25, 44, and 53 dpo), *E. multicarinata* (*N* = 3; 12, 29, and 40 dpo), and *B. capensis* (*N* = 3; 13, 23, and 41 dpo) were provided by Dr. Raul Diaz's laboratory (California State University, Los Angeles ‐ CSULA) and digitized at the Molecular Imaging Center (University of Southern California) using a Nikon XT H 225ST industrial scanner. These samples underwent a dehydration protocol using graded ethanol and methanol solutions, followed by immersion in a 90% methanol solution containing 0.7% PTA for up to 12 days, with daily solution changes. The samples were then rehydrated and stored in 0.01% sodium azide PBS solution until scanning.^58^ Embryos collected by RED received approval from the Institutional Animal Care and Use Committee (IACUC) at CSULA (#1016‐02; State of California Department of Fish and Wildlife #D‐0037819404‐6).

### 4.2 Segmentation and 3D modeling

Bronchi, tracheal, and pulmonary lumens were segmented to create three‐dimensional models, following methods described by Schachner et al.^59^ The models were analyzed qualitatively to (1) describe pulmonary anatomy and (2) investigate the presence of a multicameral stage, as suggested by Lambertz et al.^31^

Three‐dimensional surface models were generated using Avizo 2023.1.1 (Thermo Fisher Scientific) software. Semi‐automatic grayscale thresholding was combined with manual segmentation for precise isolation of airways. Thresholding parameters were adaptively adjusted to optimize contrast between soft tissues and air. Regions with insufficient contrast, such as the trachea and bronchi, were segmented manually in the axial plane. Subsequent segmentation was validated in sagittal and horizontal (dorsal) planes, reconstructed orthogonally in Avizo, and cross‐checked in the axial plane for consistency.^40^

Imaging data in DICOM (Digital Imaging and Communications in Medicine) format are available on MorphoSource: https://www.morphosource.org/projects/000752350?locale=en.
